# Tooth shape asymmetry in post-canine dentition: Evidence-based morphometric insights

**DOI:** 10.1016/j.jobcr.2025.04.010

**Published:** 2025-04-19

**Authors:** Srikant Natarajan, Junaid Ahmed, Shravan Shetty, Nidhin Philip Jose, Sharada Chowdappa, Sunita Carnelio

**Affiliations:** a Department of Oral Pathology and Microbiology, Manipal College of Dental Sciences, Mangalore, Manipal Academy of Higher Education, Manipal, India; b Department of Oral Medicine and Radiology, Manipal College of Dental Sciences, Mangalore, Manipal Academy of Higher Education, Manipal, India; c Department of Orthodontics and Dentofacial Orthopaedics, Manipal College of Dental Sciences, Mangalore, Manipal Academy of Higher Education, Manipal, India; d Private Dental Researcher, Mangaluru, India; e Department of Oral Pathology and Microbiology, Manipal College of Dental Sciences, Manipal, Manipal Academy of Higher Education, Manipal, India

**Keywords:** Geometric morphometry, Innovation, Dentistry, Tooth, Anatomy, Sexual dimorphism

## Abstract

**Background:**

This study investigates the potential existence of asymmetry in the shape of tooth and location of landmarks of tooth anatomy and its variation between sexes.

**Methods:**

Our study evaluated dental symmetry in 160 patients aged 13–20 years, focusing on post-canine dentition. Using 3D geometric morphometrics, the research evaluated the variations in the anatomical landmarks between left and right side.

**Results:**

Analysis of the landmarks revealed that 20–30 % of the principal components explained 80 % of the shape variation. No significant difference in centroid size was found between sexes, but significant shape differences were noted in all teeth except the 3-cusp type mandibular second premolar. Asymmetry was more in the premolar segment in the maxillary arch and in the two-cusp type of mandibular second premolar and the mandibular first molar in the mandibular arch.

**Conclusion:**

The research underscores the evolutionary advantage of bilateral symmetry and the presence of fluctuating asymmetry, possibly linked to genetic or environmental stressors. It emphasizes the importance of understanding dental asymmetry for effective treatment planning and diagnosis, in restorative dentistry and orthodontics.

## Graphical abstract

**Figure:**
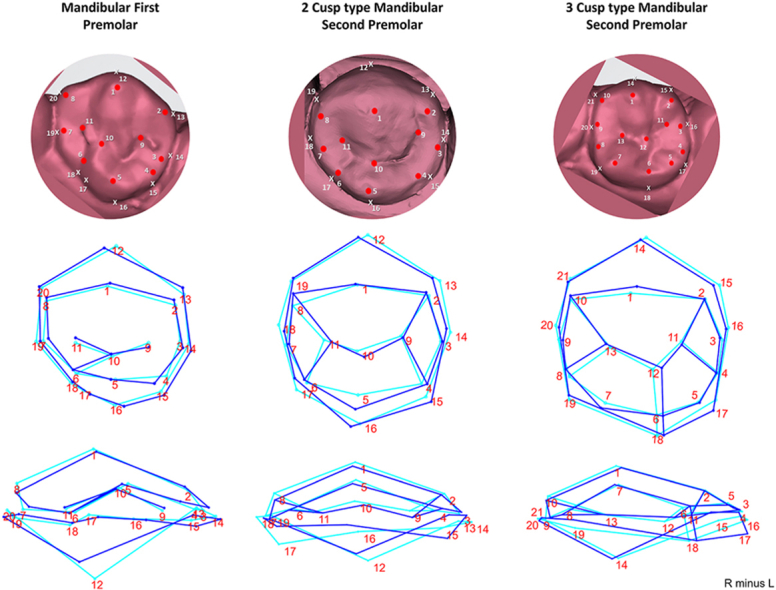

## 1 Introduction

All beings, particularly their faces, have mirror symmetry across their midline (mid-sagittal plane), with very few exceptions. The left and right teeth intraorally exhibit the exact symmetry. Very early in the development of a fetus, the body establishes symmetry across the midline. As a result, it contributes to crucial advantages from an evolutionary standpoint, like enhanced chewing and locomotor abilities. Visual experience for the patient or dentist involves the recognition and reproduction of bilaterally symmetric patterns in terms of placement of teeth or shape of tooth selected.^1^ In the maxilla and mandible of early mammals, the evolution of molars, premolars, canines, and incisors from haplodont beginnings occurred mirror-symmetrically on both sides. Two incisors, a canine, two premolars, and three molars are evenly spaced on both sides of individuals' upper and lower jaws. The eruption timing of both deciduous and permanent teeth displays bilateral symmetry with very slight variations.^2^ The development of 3D technology in the last ten years, including intraoral scanners, reasonably priced laser cast scanners, and photogrammetry, has made it feasible to assess the anatomy of teeth form and alignment in great detail.^3^, ^4^, ^5^

A random departure from symmetry is fluctuating asymmetry, which means there are small but noticeable deviations in symmetry between the left and right sides. This could be connected to a local phenomenon or environmental or genetic changes that might be widespread among a certain population. These genetic or ecological differences might be referred to as "stressors" that could impact the occurrence of a population-level fluctuating asymmetry.^6^^,^^7^ one side may be constantly different from the other in a certain way; this is known as directional asymmetry. The teeth may be asymmetrical in both size and form. Since the genetic material migrates from the same origin, it is optimal for teeth to grow on each side in mirror copies of one another. However, side variances might happen due to genetic and epigenetic causes. The size of the teeth in the permanent dentition is known to exhibit considerable variation associated with hereditary factors accounting for 30 to 95 percent. Although a similar trend is noted in the primary dentition, and it is reported that the permanent teeth have greater asymmetry.^8^ Asymmetry may indicate developmental instability or the host's reaction to temperature, nutritional, chemical, or toxicant distribution. The presence of stressors in the mother while the child is in the womb or infancy incites odontometric asymmetry.^9^ The hormonal "overflow" seen in different age groups of males and females may impact how asymmetry develops. The tooth that displays asymmetry may vary depending on whether a person is male or female because of the timing of development spurts.^8^

For proper treatment planning and diagnosis, it's crucial to be cognizant of the degree of asymmetry in the size of the teeth, jaw, and soft tissues. It is equally important to be able to distinguish between "pathological" and "normal" asymmetry. Asymmetry in the masticatory system, which includes the muscles of the tongue, masticatory apparatus, and pharyngeal muscles, may affect how well the deglutition process works, how well you can breathe, and how well your jaw develops its ideal occlusion. Because they can cause asymmetry in the teeth and occlusion, parafunctional activities and changes in the temporomandibular joint must be carefully considered.^7^ Leigh Van Valen in 1962 was the first to study dental asymmetry in the dimensions of horse teeth.^10^ Researchers have reported asymmetry in the size of the hominid teeth including human teeth.^8^^,^^9^^,^^11^

A review of the literature indicates that craniofacial asymmetry has been extensively studied and correlated with growth patterns and tooth function.^7^ However, the asymmetry in tooth morphology between the left and right sides of the same individual remains an unexplored research gap. While three-dimensional analyses have been conducted on facial asymmetry,^12^^,^^13^ similar investigations into tooth shape asymmetry have not been reported. With this research, we aim to document and analyze the morphological differences in tooth shape between the left and right sides of the human postcanine dentition, addressing this gap in the existing body of knowledge.

The current study evaluated post-canine dentition (maxillary and mandibular, first and second, premolars and molars) for shape asymmetry. The location of the cusps, ridges and crests of curvature will affect how occlusion is developed and restorations or reconstruction of tooth is done. The presence of asymmetry may influence not only the occlusion but also the homeostasis of the orofacial region's jaw, temporomandibular joint, and neuromuscular coordination. We hypothesized that asymmetry could exist in the shape and placement of the cusps, marginal ridges, and crests of curvatures of the teeth, even if there were not significant differences in tooth size between the left and right sides.

## 2 Materials and methods

Following informed written consent from the patients to use the dental impressions for research purposes, pretreatment dental impressions (n = 120) were collected using rubber-based impression material, from the Department of Orthodontics, Manipal College of Dental Sciences, Mangalore, Karnataka, India. The sample size was derived based on the study of mesiodistal asymmetry reported by Naseri N et al.^14^ Sample size calculation was done using sample size calculation for two means, where N=(2[[(Z_1-α/2_+ Z_1-β_)^2^]σ^2^]/d^2^), where, N is the sample size, σ refers to the average standard deviation, and d refers to the clinically significant difference. The standard deviations reported were 0.5090 and 0.9210 for the mesiodistal dimensions of the left and right maxillary first molars, respectively (from Table 1 of Naseri N et al., 2016 study). This tooth was selected as it showed the least asymmetry in the study.^14^ Keeping an alpha error of 5 % (corresponding to a z value of 1.9599) and 80 % power (corresponding to a z value of 0.842) and a clinically significant difference of 0.4 units, the required sample in each group was derived as 51. We took a sample of 120 pretreatment casts for 60 males and 60 females.

**Table 1:** Procrustes ANOVA utilized to evaluate centroid size and shape between sexes and side in each teeth.

		Sex				Side				Sex ∗ Side			
		SS	MS	F	P	SS	MS	F	P	SS	MS	F	P
Maxillary First Premolar	Centroid	0.195015	0.195015	0.51	0.6055	0.042903	0.042903	0.11	0.7944	0.383241	0.383241	0.49	0.4827
	Shape	0.031022	0.000585	2.81	**0.0001**	0.055519	0.001048	5.03	**<.0001**	0.011044	0.000208	0.76	0.9038
Maxillary Second Premolar	Centroid	0.164805	0.164805	231.59	0.0418	0.048852	0.048852	68.65	0.0765	0.000712	0.000712	0	0.9752
	Shape	0.023055	0.000461	4.94	**<.0001**	0.05638	0.001128	12.08	**<.0001**	0.004668	9.34E-05	0.4	0.9999
Maxillary First Molar	Centroid	8.356823	8.356823	244.47	0.0407	0.126196	0.126196	3.69	0.3055	0.034184	0.034184	0.03	0.8719
	Shape	0.031533	0.00041	1.93	**0.0022**	0.05205	0.000676	3.18	**<.0001**	0.016366	0.000213	0.8	0.894
Maxillary Second Molar	Centroid	13.48466	13.48466	117.91	0.0585	0.301145	0.301145	2.63	0.3516	0.11436	0.11436	0.02	0.8796
	Shape	0.048288	0.000627	2.44	**<.0001**	0.051653	0.000671	2.61	**<.0001**	0.019805	0.000257	0.78	0.9227
Mandibular First Premolar	Centroid	0.011416	0.011416	0.26	0.701	0.045974	0.045974	1.04	0.4942	0.044319	0.044319	0.06	0.8102
	Shape	0.033466	0.000631	2.32	**0.0013**	0.089273	0.001684	6.2	**<.0001**	0.014394	0.000272	0.52	0.9986
2 Cusp Type Mandibular Second Premolar	Centroid	0.096101	0.096101	0.25	0.7055	0.153108	0.153108	0.4	0.6424	0.38654	0.38654	0.38	0.5398
	Shape	0.039882	0.000798	3.31	**<.0001**	0.038081	0.000762	3.16	**<.0001**	0.012038	0.000241	0.59	0.9912
3 Cusp Type Mandibular Second Premolar	Centroid	0.07679	0.07679	24.73	0.1263	0.213203	0.213203	68.65	0.0765	0.003106	0.003106	0	0.953
	Shape	0.027197	0.000486	1.33	0.1417	0.04489	0.000802	2.2	**0.0018**	0.020381	0.000364	0.98	0.5238
Mandibular first Molar	Centroid	2.416773	2.416773	40.13	0.0997	0.200454	0.200454	3.33	0.3192	0.060225	0.060225	0.04	0.8474
	Shape	0.025398	0.000285	4.34	**<.0001**	0.049095	0.000552	8.38	**<.0001**	0.005859	6.58E-05	0.55	0.9998
Mandibular Second Molar	Centroid	11.71446	11.71446	10.28	0.1925	0.451295	0.451295	0.4	0.6425	1.140062	1.140062	0.54	0.465
	Shape	0.037822	0.000511	4.64	**<.0001**	0.050362	0.000681	6.18	**<.0001**	0.008156	0.00011	0.56	0.9993

The patient cohort for the study consisted of participants between the ages of 13 and 20 years. The person's domicile, place of birth of their parents and grandparents, and a complete demographic background were all recorded. Individuals were classified as being from the Dakshina Kannada region if they had a lineage of three generations. People with dental caries, a history of trauma, or restorations of their mandibular posterior teeth (First Premolar to Second Molar) were excluded from participating in the study. Furthermore, patients with developmental anomalies that may affect the size or shape of their teeth and subjects with signs of syndromes associated with tooth shape/structure were excluded from the study.

After the impressions were taken and poured, the casts were digitalized using the inEOS X5-Lab scanner (Dentsply Sirona, India). The inEos X5-Lab scanner utilizes digital light stripe projection to achieve high-precision scanning of dental cast models. It has a five-axis robotic arm which allows for flexible positioning, ensuring comprehensive coverage of complex geometries. Based on the anatomical and geometrical characteristics of the tooth, we determined the essential characteristics that most accurately described the landmarks following the research on describing the basal region of posterior teeth by Biggerstaff R (1969).^15^

We defined the landmarks on the tooth based on the geometric and anatomic evidences on the post-canine dentition by reviewing the landmarks proposed by Robinson DL et al. (2002) and Al-Shahrani I et al. (2014).^16^^,^^17^

Geometric evidence (based on the crests of curvature, line and point angles, surface landmarks corresponding to the occlusal surface landmarks) and anatomic evidence (corresponding to cusp tips, fissure junctions, endpoints of ridges, grooves and fissures) were used to identify the landmarks. Slicermorph software (http://www.slicer.org), was used to annotate the 3D models of these sites.^18^ Depending on the complexity of the tooth morphology, the number of landmarks ranged from 19 to 32. The supplementary file provides detailed information on these landmarks and includes eight tables, each corresponding to a specific tooth. (Supplementary file) For the form analysis, 120 digital models that demonstrated the presence of maxillary and mandibular first premolar to second molars bilaterally were chosen. The casts of all 60 males and 60 females had first premolars, first molars, and second molars. The mandibular second premolars in 37 of the 120 patients had two cusps, while the second premolars in 83 of the patients had three cusps. To increase the accuracy of our analysis of shape of the two morphological variants of mandibular second premolar, we included 40 more casts. This allowed us to look into the shape variation of the two (n = 49) and three cusp type (n = 111) mandibular second premolars. The landmark coordinates were recorded and reformatted in Microsoft Excel (Microsoft Corporation, 2023) and saved in ∗.csv format. This file was then imported into MorphoJ, an open-source integrated program package for geometric morphometrics (version 1.08.01, for Microsoft Windows, Klingenberg, C. P. 2011, https://morphometrics.uk/MorphoJ_page.html).^19^

The landmarks were then normalized using generalized procrustes superimposition technique. Principal component analysis was used on the normalized landmarks in order to assess their role in shape variation. Procrustes ANOVA was performed to evaluate the symmetry between left and right side landmark data, incorporating sex as a variable to test for interaction.

## 3 Results

The landmarks were obtained from the 120 digitized casts (constituting 60 males and 60 females), of individuals from Dakshina Kannada, Mangaluru, region of Karnataka, India. The principal component analysis revealed that 20–30 % of the principal components—which account for 11–23 components based on the teeth—explained 80 % of the variation in shape. The Procrustes ANOVA test was used to assess the differences in tooth shape between sexes, followed by the analysis of symmetry between the sides. According to Procrustes ANOVA, the centroid size between the sexes did not significantly differ in all teeth. Centroid size is calculated as “the square root of the sum of squared distances” from all landmarks to their geometric center (centroid). In geometric morphometry, centroid size refers to a scale-independent measure of size. It represents the overall size of a structure without influencing its shape. However, the shape is significantly different between the sexes in all teeth except the 3 cusp type mandibular second premolar. Additionally, all variations reported in the results pertain to differences between the left and right sides. There was no significant interaction between sex and side in shape and centroid size, indicating that asymmetry does not differ between males and females (Table 2). The percentage of variation indicated by side ranges from 0.845 % in the maxillary second molar to 2.007 % in the maxillary second premolar. Mandibular second premolar (3 cusp type) shows the least asymmetry among the teeth in the mandibular arch. Mandibular second premolar (2 cusp type) demonstrated the highest variation between the sides as well as among the sexes accounting for 1.945 % and 2.037 % respectively. Asymmetry is noted more in the premolar segment in the maxillary arch and in the two cusp type of mandibular second premolar and the mandibular first molar in the mandibular arch (Table 3).

**Table 2:** Percentage of sum of squares explained in relation to total in centroid size and shape as explained by procrustes ANOVA.

	Centroid Size				Shape			
	Total ss	SS Explained by Sex (P value)	SS Explained by Side (P value)	SS expained by Sex∗Side (P value)	Total ss	SS Explained by Sex (P value)	SS Explained by Side (P value)	SS expained by Sex∗Side (P value)
Maxillary First Premolar	99.66164115	0.106(0.6055)	0.023(0.7944)	0.209(0.4827)	3.54248729	**0.876(0.0001)**	**1.567(<.0001)**	0.312(0.9038)
Maxillary Second Premolar	99.87642904	0.095(0.0418)	0.028(0.0765)	0(0.9752)	2.80872433	**0.821(<.0001)**	**2.007(<.0001)**	0.166(0.9999)
Maxillary First Molar	97.32318564	2.626(0.0407)	0.04(0.3055)	0.011(0.8719)	4.90263053	**0.643(0.0022)**	**1.062(<.0001)**	0.334(0.894)
Maxillary Second Molar	98.83004439	1.135(0.0585)	0.025(0.3516)	0.01(0.8796)	6.11265649	**0.79(<.0001)**	**0.845(<.0001)**	0.324(0.9227)
Mandibular First Premolar	99.94381351	0.006(0.701)	0.025(0.4942)	0.024(0.8102)	6.69277583	**0.5(0.0013)**	**1.334(<.0001)**	0.215(0.9986)
2 Cusp Type Mandibular Second Premolar	99.32014352	0.103(0.7055)	0.164(0.6424)	0.413(0.5398)	1.95820907	**2.037(<.0001)**	**1.945(<.0001)**	0.615(0.9912)
3 Cusp Type Mandibular Second Premolar	99.84931693	0.039(0.1263)	0.11(0.0765)	0.002(0.953)	4.64032154	0.586(0.1417)	**0.967(0.0018)**	0.439(0.5238)
Mandibular first Molar	99.30595227	0.626(0.0997)	0.052(0.3192)	0.016(0.8474)	2.57611183	**0.986(<.0001)**	**1.906(<.0001)**	0.227(0.9998)
Mandibular Second Molar	97.4201683	2.271(0.1925)	0.088(0.6425)	0.221(0.465)	3.55629708	**1.064(<.0001)**	**1.416(<.0001)**	0.229(0.9993)

Bold values indicate statistically significant association.

**Table 3:** Overview of morphological variations noted in tooth outlines between left and right sides, analyzed from XY axis (showing mesiodistal and buccolingual variations) and XZ axis (showing cervico-occlusal variations).

Tooth	Landmarks	Aspect	Morphological Asymmetry Observed
Maxillary First Premolar	2, 3, 4	Distal Marginal Ridge	Thickness variation
	13, 17	Buccal and Lingual Crests of Curvature	Mesiodistal and cervico-occlusal asymmetry
	12–7	Mesial Marginal Developmental Groove	Relatively stable
	11-10-12	Central Groove	Relatively stable
Maxillary Second Premolar	2, 3	Buccal half of distal marginal ridge	Mesiodistal asymmetry
	7	Position and thickness of mesial marginal ridge	Mesiodistal asymmetry with variation in thickness
	5	Lingual Cusp Tip	Cervico-occlusally similar, buccolingual asymmetry
	16, 12	Buccal and Lingual Crests of Curvature	Mesiodistal asymmetry noted, buccal crest shows more mesial and lingual crest shows more distal positioning
Maxillary First Molar	1	Mesiobuccal Cusp	Asymmetry observed in buccolingual and mesiodistal direction
	9	Mesiolingual Cusp	Asymmetry observed in buccolingual and mesiodistal direction
	21	Distal Crest of Curvature	Asymmetry observed in buccolingual and mesiodistal direction
	14	Midpoint of the Transverse groove of Oblique Ridge	Asymmetry observed in buccolingual and mesiodistal direction
	4, 5, 6	Outline of the occlusal table formed by the distal marginal ridge	Asymmetry in distal outline in mesiodistal direction
	14-15-16	Central Groove	Buccolingual asymmetry
	8–24	Lingual Groove	Buccolingual and mesiodistal asymmetry
	18, 24	Buccal and Lingual Groove Ends	Cervico-occlusal asymmetry
Maxillary Second Molar	17–22	Distobuccal Outline	Asymmetry observed in the distobuccal-mesiolingual direction
	13–16	Central Groove	Buccolingual variation noted but cervico-occlusally stable
	9	Mesiolingual Cusp Tip	Buccolingual asymmetry
	25	Crest of Curvature associated with mesiolingual cusp	Buccolingual asymmetry
	8–24	Lingual Groove	Mesiodistal asymmetry
	28	Mesiobuccal line angle	Cervico-occlusal and buccolingual variation
	12	Buccal end of mesial marginal ridge	Cervico-occlusal and Mesiodistal variation
	21	Distal crest of curvature	Cervico-occlusal and Mesiodistal variation
Mandibular First Premolar	12, 16	Buccal and Lingual Crests of Curvature	Mesiodistal asymmetry
	4	End of Distal Slope of Lingual Cusp	Mesiodistal asymmetry
	1, 5	Buccal and Lingual Cusp Tips	Minimal variation
	6–10	Mesiolingual Groove	Mesiobuccal-distolingual variation
	12, 8	Buccal Crest of Curvature and Mesial End of Mesial Cusp Slope	Cervico-occlusal variation
Mandibular Second Premolar 2 Cusp type	5	Lingual Cusp Tip	Buccolingual asymmetry
	3–14	Mesial Marginal Ridge	Width variation
	9	Mesial Pit	Cervico-occlusal variation
	8	Distal End of Distal Slope of Buccal Cusp	Buccolingual asymmetry
	1, 5	Cusp Heights	Cusp height variation
	18	Distal Contact Point	Cervico-occlusal stability, distal placement variation
	15, 17	Mesiolingual and Distolingual Line Angles	Cervico-occlusal and buccolingual asymmetry
	12	Buccal Crest of Curvature	Cervico-occlusal and buccolingual asymmetry
Mandibular Second Premolar 3 Cusp type	7, 5	Mesiolingual and Distolingual Cusps	Size variation
	12-6-18	Lingual Groove	Mesiodistal variation
	14–15	Distal Cusp Slope of Buccal Cusp	Variation observed in the distobuccal-mesiolingual direction
	17	Distolingual Line Angle	Mesiodistal and cervico-occlusal variation
	2,3,4,5	Distal outline of the occlusal table	Relatively stable
Mandibular First Molar	20, 22, 23	Buccal Outline	Relatively stable
	3,4,5,6,7	Distal Outline of Occlusal Table	Relatively stable
	22,23,24,25,26	Distal Outline of Tooth	Relatively stable
	19, 18	Mesial Pit, Central Pit	Mesiodistal asymmetry
	2–21	Mesiobuccal Groove	Mesiodistal asymmetry
	27, 28, 29, 30	Lingual and Mesiolingual Contour	Buccolingual asymmetry
	1, 5	Mesiobuccal and Distal Cusp Tips	Cervico-occlusal asymmetry
	3, 11, 9	Distobuccal cusp tip, Msiolingual and distolingual cups tip	Mesiodistal and cervico-occlusal asymmetry
	21, 28	Mesiobuccal and Distobuccal Grooves	Vertical variation in cervicoocclusal axis
Mandibular Second Molar	1	Mesiobuccal Cusp Tip	Buccolingual and cervico-occlusal asymmetry
	9	Mesiolingual Cusp Tip	Mesiodistal asymmetry
	14	Central Pit	Mesiodistal asymmetry
	15, 13	Mesial and Distal Pits	Mesiodistal and vertical variation
	24	Mesiolingual Crest of Curvature	Asymmetry observed in buccolingual direction

**Fig. 1:**
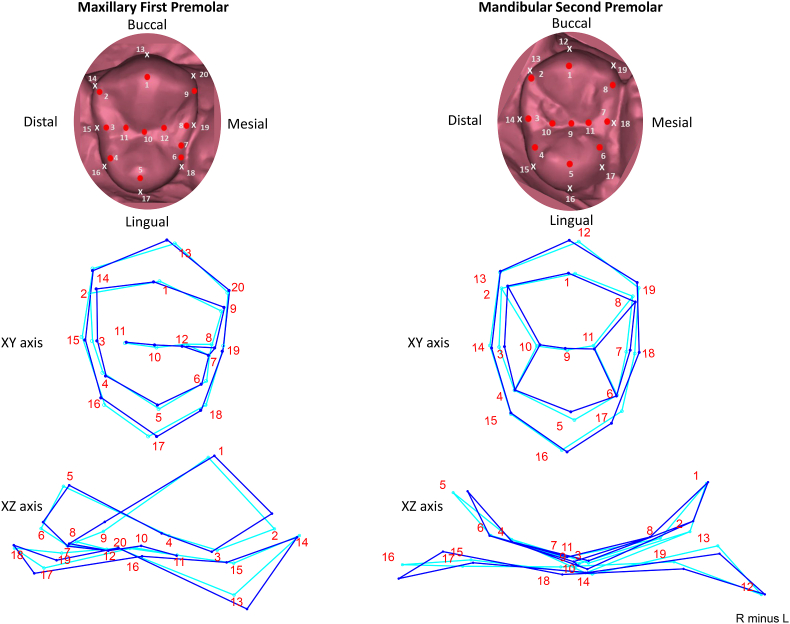
Variation in the landmarks in Maxillary first and second premolar. A wireframe graph representing change from right (indicated by dark blue lines) to left (indicated by light blue lines). (For interpretation of the references to colour in this figure legend, the reader is referred to the Web version of this article.)

The maxillary first premolar exhibits asymmetry in multiple aspects. The distal outline (landmark 14–16) shows mesiodistal variation, and the thickness of the distal marginal ridge (landmark 3, 15) may differ between the left and right sides. The position of the buccal and lingual crests of curvature (landmark 13 and 17) varies mesiodistally or cervico-occlusally. However, the location of the mesial marginal developmental groove (landmark 7.12) and the central groove (landmark 11-10-12) remains relatively stable when comparing the left and right sides. The maxillary second premolar is relatively stable in its distal outline (landmarks 13–15), showing minimal variation. However, the mesial marginal ridge position (landmarks 18 and 7) tends to be more mesially placed when compared between the sides. Other landmarks exhibiting variation include the lingual cusp tip (landmark 5) which remains cervico-occlusally similar but shows asymmetry in the buccolingual and mesiodistal directions. The lingual and buccal crests of curvature (landmark 16 and 12) also exhibit mesiodistal asymmetry, with the lingual crest of curvature additionally showing variation along the cervico-occlusal direction (Fig. 1).

**Fig. 2:**
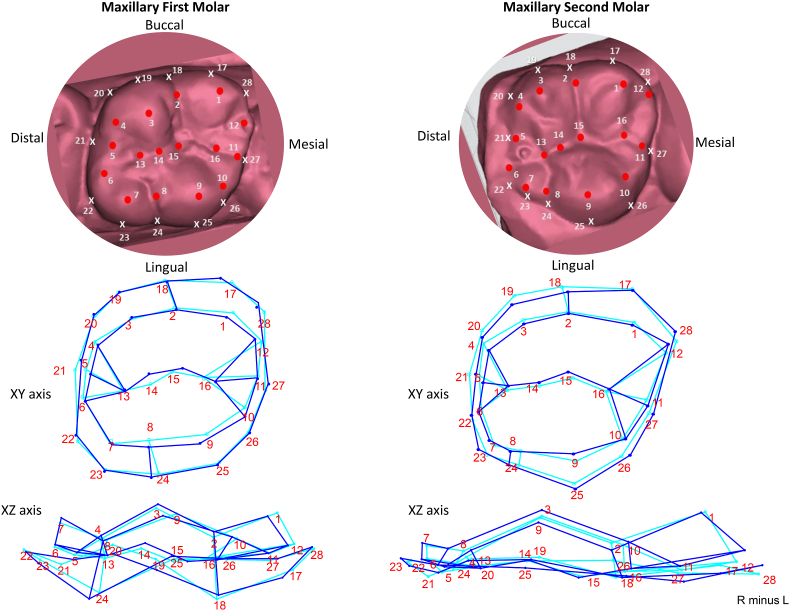
Variation in the landmarks in Maxillary first and second molar. A wireframe graph representing change from right (indicated by dark blue lines) to left (indicated by light blue lines). (For interpretation of the references to colour in this figure legend, the reader is referred to the Web version of this article.)

The maxillary first molar displays asymmetry in the mesiobuccal cusp (landmark 1), mesiolingual cusp (landmark 9), distal crest of curvature (landmark 21), and the midpoint of the transverse groove of the oblique ridge (landmark 14), with variations in both the buccolingual and mesiodistal directions. The cervico-occlusal axis remains relatively stable, but the greatest variation is observed in the mesiobuccal cusp (landmark 1) and the end of the lingual groove (landmark 24), which exhibit both mesiodistal and buccolingual asymmetry. The central groove (landmarks 13–16) pattern is irregular and shows buccolingual positional variation, while the central pit (landmark 15) exhibits mesiodistal variation. The maxillary second molar demonstrates asymmetry in the size of the occlusal table due to the variation in the distobuccal outline (landmarks 17–20), which tends to be wider in the distobuccal region. The central groove pattern (landmarks 13–16) remains consistent on both sides but displays buccolingual positional asymmetry (Fig. 2).

**Fig. 3:**
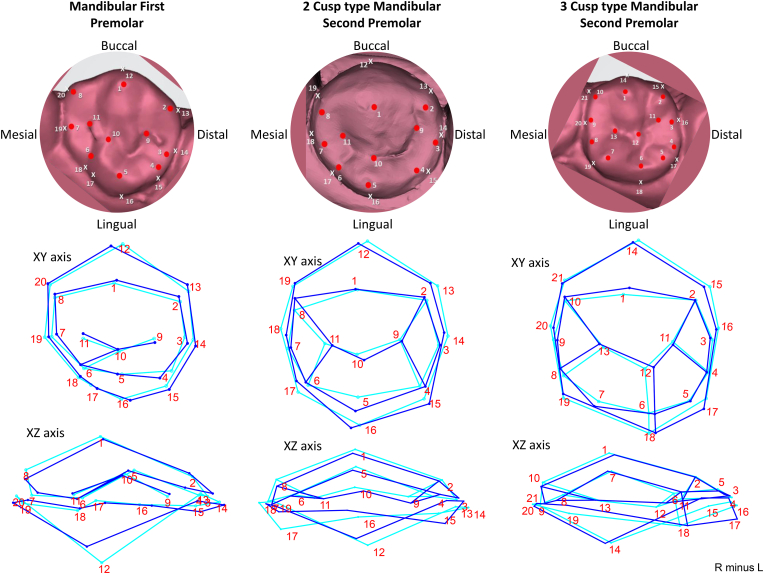
Variation in the landmarks in Mandibular Premolars. A wireframe graph representing change from right (indicated by dark blue lines) to left (indicated by light blue lines). (For interpretation of the references to colour in this figure legend, the reader is referred to the Web version of this article.)

The mandibular first premolar showed that the buccal and the lingual crests of curvature (landmark 12 and 16), end of the distal slope of the lingual cusp (landmark 2) show the greatest variation mesiodistally indicating the positioning of the landmarks vary in mesiodistal axis. The buccal and the lingual cusp tips (landmark 1 and 5) are relatively stable showing minimal variation. The location of the termination of the mesiolingual groove occlusally shows variation in the mesiobuccal-distolingual aspect. (landmark 6) In the vertical dimension the location of the buccal crest of curvature (landmark 12) and the mesial end of the mesial cusp slope of the buccal cusp (landmark 8) show the greatest variation cervico-occlusally (Fig. 3).

Comparison of the symmetry of the mandibular second premolar (2 cusp type) shows that variations are noted in the bucco-lingual position of the lingual cusp tip (landmark 5), width of the mesial marginal ridge (landmark 7 and 18) and location of the mesial pit (landmark 11). The distobuccal cusp slope (landmark 1–2) may be aligned parallel to each other but not the mesial (landmark 1–8). The cusp heights (landmark 1 and 5) and the level of contact points (landmark 14 and 18) are more constant however the maximum variation are noted in the mesiolingual line angle followed by the height of the buccal crest of curvature (landmark 12). Comparison of the symmetry of the mandibular second premolar (3 cusp type) shows maximum variation in the size of the mesiolingual cusp(polygon bounded by landmarks 6-7-8-13-12) and distolingual (polygon bounded by landmarks 11-12-6-5-4) cusps in terms of size and mesiodistal location. The lingual groove (landmark 12-6) varies in its location mesiodistally. The variation is minimal in terms of the cervical crest of curvature (landmark 14 and 18). The maximum variation is seen in relation to the distal lingual line angle (landmark 17) (Fig. 3).

**Fig. 4:**
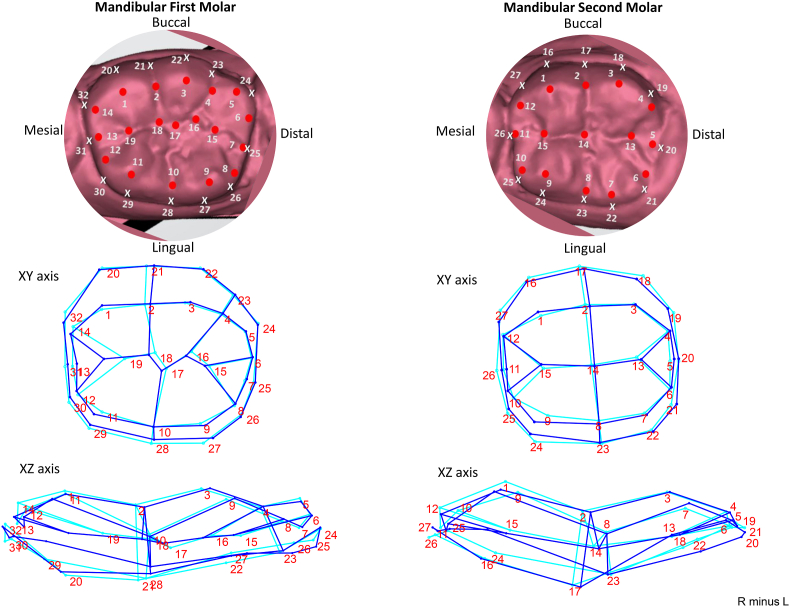
Variation in the landmarks in Mandibular first and second molar. A wireframe graph representing change from right (indicated by dark blue lines) to left (indicated by light blue lines). (For interpretation of the references to colour in this figure legend, the reader is referred to the Web version of this article.)

The mandibular first molar's occlusal outline is stable with minimal variation in the occlusal table or the external outline between the left and right sides. Maximum asymmetry is observed in relation to the mesial pit (landmark 19) in the mesiodistal direction. The second molar's also is relatively stable in shape. The occlusal outline shows maximum asymmetry in relation to the mesiolingual cusp tip (landmark 9) in buccolingual and mesiodistal diecction. The tooth may be bulkier in relation to the mesiolingual crest of curvature (landmark 24) (Fig. 4).

Table 3 summarizes the asymmetries identified in the post-canine dentition of both the maxillary and mandibular arches. These observations are made from two aspects: the mesio-distal and bucco-lingual aspect (x-y axis) and the cervico-occlusal aspect (x-z axis).

## 4 Discussion

The occurrence of asymmetry can be attributed to physiological processes, muscle movements, behavioural patterns, environmental factors, disruptions in development, genetic and hormonal influences. Asymmetry is a fundamental feature of biological structures, which may be directional or fluctuating, the latter being more common. Fluctuating asymmetry may be attributed to uneven distribution of transcription factors in the developing tissues, problems in molecular levels' activation mechanisms, or excessive molecular signals leading to noise at a molecular level.^20^ The study by Rolfe S et al. (2018), indicates NFATC1, SOX5, and TCF7L1 are significant transcription factors which influence facial asymmetry by modulating craniofacial, skeletal and through Wnt signalling pathways respectively.^21^

Facial asymmetry affects orofacial function and appearance, with its impact varying based on individual functional and psychological factors. Tooth shape asymmetry has both functional and esthetic implications, requiring an understanding of 3D and sectional anatomy to assess normal variation. Advances in **3D data acquisition** through optical scanning, photogrammetry, and radiographs coupled with the rise of open source morphometric softwares, have improved asymmetry analysis, have enabled us to gain fresh insights, by identifying new correlations and interactions among multiple parameters determining shape change, asymmetry and sexual dimorphisms.^22^ We have evaluated the asymmetry of human post-canine dentition, based on geometric and anatomic landmarks using geometric morphometry.

Garn et al. reported that distal tooth of a particular class (i.e. Lateral incisor, second premolar or second molar) tends to show higher degree of asymmetry.^23^ The distal extension of the dental lamina, which creates the first molar, is known to give rise to the second and third molars. Therefore, the second and third molar sizes are influenced by the size of the first molar.^24^ This hypothesis is true in **our study** concerning maxillary premolars, where the second premolar shows greater asymmetry than the first premolar. Due to two morphologically distinct mandibular premolars, similar interpretations are inappropriate for the mandibular arch. Understanding the variance in premolar form and cusp number in relation to the evolution of dentition in primates and hominins is essential. All mammals exhibit heterodonty, meaning they have multiple teeth classes, in humans, four teeth classes: incisors, canines, premolars, and molars. The dental formula in placental animals, or prenatal mammals with a placental connection for the kid, was 5-1-4-3, or 5 incisors, 1 canine, 4 premolars, and 3 molars. The pattern in evolution was a decrease in tooth count and an increase in the number of cusps on posterior teeth.^25^ Prehistoric hominins had pointed premolars, but they developed multicuspid teeth to fit their herbivorous diets as they evolved. No living primates have four premolars; primitive primates, tarsiers, and New World monkeys have three on each side of each jaw. Apes, old world monkeys and humans have only 2 premolars in each quadrant and share the dental formula 2-1-2-3.^26^

Although, humans have two mandibular premolars,the mandibular second premolars exhibit "molarization", which is described as increase in size, increase in cusps or change in function.^27^^,^^28^ The 3 cusp type mandibular second premolar is the distally placed premolar, can be considered as the more variable one as they exhibit "Molarization". **In our study** however, the three-cusp type mandibular second premolar was found to be relatively shape stable and exhibited the least asymmetry in the teeth of the mandibular arch. This is quite a unique result, as it is noted in previous studies that the more distally placed type of teeth in a particular class exhibits more asymmetry.^14^^,^^23^^,^^29^^,^^30^ **Our findings** show that the maxillary arch in the premolar shape region corresponds with Garn et al.'s theory (distal tooth shows more asymmetry), while the mandibular arch does not.^23^ It is quite possible to achieve such a result because the premolars develop independently from the zone that influences the formation of molars. Our research revealed that the mandibular second premolar, which has two cusps, exhibits more asymmetry than the mandibular first premolar. In an earlier investigation of fluctuating asymmetry in the South African population, Groeneveld and Kieser discovered such a reversal in the symmetry pattern, i.e. they found the maxillary second premolar more asymmetric than the maxillary first premolar but the pattern was reversed in the mandibular premolar zone where in the first premolar was more asymmetric. This is similar to our results. They also demonstrated that the shape asymmetry predominates in the maxillary post-canine dentition, but size asymmetry predominates in the mandibular post-canine dentition.^31^

The hypothesis of the distal tooth of a particular class showing greater asymmetry doesn't hold true for both maxillary and mandibular molars. In our study, when the molar segment was examined, it was discovered that the first molar in both the maxilla and the mandible had higher asymmetry than the second molar. The first molars in both arches showed higher degree of asymmetry than the second molars. Study by Barret et al. on the shape of the molars showed similar asymmetry pattern in both Neanderthals and modern humans.^20^ Study of occlusal polygon area of maxillary and mandibular molars by Garcia et al. (2022) in Colombian population showed significant asymmetry between the left and the right first molars.^32^ Garn et al. had observed that the asymmetry of the tooth correlated moderately with the size of the tooth, i.e. the larger the tooth the greater would be the variation in the left and right side characteristics leading to higher chances of asymmetry.^23^ However, our results found that the maxillary and mandibular first molars (the largest teeth in the quadrant) were relatively stable and showed the least asymmetry. This singularity can be attributed to genetic and environmental factors influencing development of teeth. First, molars show the first evidence of calcification and crown formation before birth and during early infancy. Environmental changes during pregnancy and the first few years of life may function as "stressors" and cause more developmental instability. Leamy et al. (2005) investigated the relationship between mouse genes for molar size and shape and epistatis, a type of gene interaction in which one gene may dominate the phenotypic expression character. He discovered that mutations in gene loci on chromosomes 9 and 11 were significantly associated with molar shape without significant change in its size.^33^

The differences in the tooth shapes on the left and right sides have very clear clinical implications. First, decisions on premolar extraction for orthodontic therapy become more critical if significantly asymmetrical premolar forms are present.^34^ Second, the asymmetry of the teeth may significantly affect the amount of space needed for orthodontic treatment as well as the amount of space that is accessible. The amount of tooth movement necessary on either side of a molar that has more tooth material on one side may change dramatically, necessitating an asymmetrical treatment plan. Thirdly, a tooth with a squarish shape will have more torque and rotation resistance and may be more challenging to realign. Finally, if the cusp forms are asymmetric, it could be challenging to achieve accurate intercuspation.^34^ The excessive crown variations on one side may cause eruption issues and affects tooth alignment unilaterally. As a result, there are inherent challenges in extracting these teeth. The tooth anatomy, variation in crown shape, locations of landmarks and groove patterns may influence the development and progress of caries which will alter the way we prevent, restore and replace teeth.^35^ Comprehending the shape of teeth from occlusal, buccal, or proximal perspectives is vital for a dentist. Dental restorations, such as fillings, crowns, bridges, and other prosthodontic or restorative treatments involving occlusion development, are influenced by the shape of the tooth. Dentists must be aware of potential asymmetries within a tooth and between opposite sides of the jaw to ensure accurate restoration.

One potential limitation of this study is the lack of comparative literature to assess trends in asymmetry over time. To the best of our knowledge this is the first study depicting the asymmetry of human postcanine dentition using 3D geometric morphometry. Further, many studies use different landmarks to evaluate shape, and this variability in landmark selection across studies makes direct comparisons of shape analysis challenging. Additionally, this is a unicentric study conducted within our research institute, and a multicentric study using standardized landmarks could provide more meaningful and generalizable data. Further research on asymmetry in twins may offer deeper insights into the true genetic influence on shape asymmetry.

## 5 Conclusion

Our research findings align with Garn et al.'s theory which states that distal teeth within a specific class are more likely to exhibit a higher degree of asymmetry. This pattern was evident in the maxillary premolars and molars, as well as the mandibular molars, where the second premolar and second molar demonstrated more asymmetry than their preceding counterparts. However, two distinct types of mandibular premolars prevent similar interpretations for the mandibular arch. It's crucial to comprehend the variations in premolar form and cusp count in the context of primate and hominin dental evolution. Our study enhances this understanding and sheds light on its implications for planning and executing restorative, prosthodontic and orthodontic treatments. This study contributes to understanding these variances and their implications for orthodontic treatment planning and execution.

## Ethics approval and consent to participate

Prior to the commencement of the study, we obtained approval from the institutional ethics committee of Manipal College of Dental Sciences Mangalore, as evidenced by reference number 20018. We also ensured to obtain written consent from the patients for the use of their dental impressions and casts for research purposes, with a firm promise of maintaining their anonymity.

## Consent for publication

Not applicable.

## Availability of data

The datasets used and/or analyzed during the current study are available from the corresponding author on reasonable request.

## Authors' contributions

SN and JA conceptualized, designed study and got funding. NPJ and SS contributed to data collection, acquiring digital data, table generation. SC1 and SC2 acquired the digital data and landmarks. SN and JA wrote the main manuscript. SN did the analysis, figures and tables, SC1,SC2 were the second observer. All authors reviewed the manuscript.

## Funding

The research is funded by the Science and Engineering Research Board (SERB, Department of Science and Technology, Government of India (file number CRG/2020/001057)

## Declaration of competing interest

The authors declare the following financial interests/personal relationships which may be considered as potential competing interests: Srikant Natarajan reports financial support was provided by 10.13039/501100001843Science and Engineering Research Board (10.13039/501100001843SERB), Government of India. If there are other authors, they declare that they have no known competing financial interests or personal relationships that could have appeared to influence the work reported in this paper.
