# Supplementary material for: Tooth shape asymmetry in post-canine dentition: Evidence-based morphometric insights
Source: J Oral Biol Craniofac Res. 2025 Apr 19;15(4):703–11. doi: 10.1016/j.jobcr.2025.04.010 (PMC12033903; doi:10.1016/j.jobcr.2025.04.010)
Supplement: Multimedia component 1 [file mmc1.docx]

# Description of Landmarks based on anatomic and geometric evidences

| Maxillary first Premolar |  |
| --- | --- |
| Landmark by Anatomic Evidence | Landmarks by Geometric Evidence |
| 1.Tip of buccal cusp: The point joining the Mesial and distal slopes of buccal cusp and buccal triangular ridge. | 13. Buccal crest of curvature |
| 2.Buccal end of distal marginal ridge : The point Junction of distal slope of buccal cusp and distal marginal ridge | 14. Distobuccal line angle |
| 3.Distal marginal ridge: One point at the mid point of the the distal marginal ridge | 15. Point on the Distal outline corresponding to the midpoint of the Distal marginal ridge |
| 4.Lingual end of distal marginal ridge: Junction of distal slope of lingual cusp and distal marginal ridge | 16. Distolingual line angle |
| 5.Tip of lingual cusp: Mesial and distal slopes of lingual cusp and triangular ridge | 17. Lingual crest of curvature |
| 6.Lingual end of Mesial marginal ridge: Junction of mesial slope of lingual cusp and mesial marginal ridge | 18. Mesiolingual line angle |
| 7.Mesial Marginal ridge: Mid point of the mesial marginal ridge | 19. Point on the Mesial outline corresponding to the midpoint of the Mesial marginal ridge |
| 8. Mesial marginal Developmental (MMD) groove: landmark point where the MMD groove crosses the mesial marginal ridge | 20. Mesiobuccal line angle |
| 9.Buccal end of the mesial marginal ridge: Junction of mesial slope of buccal cusp and mesial marginal ridge |  |
| 10.Central groove point: junction of the buccal and lingual triangular ridges |  |
| 11.Distal triangular Pit: Junction of the central groove and the distal triangular fossa |  |
| 12.Mesial triangular Pit: Junction of the central groove and the mesial triangular fossa |  |

| Maxillary Second Premolar |  |
| --- | --- |
| Landmarks by Anatomic Evidence | Landmarks by Geometric Evidence |
| 1.Tip of buccal cusp: The point joining the Mesial and distal slopes of buccal cusp and buccal triangular ridge. | 12. Buccal crest of curvature |
| 2.Buccal end of distal marginal ridge: Junction of distal slope of buccal cusp and distal marginal ridge | 13. Distobuccal line angle |
| 3.Distal marginal ridge: midpoint of the distal marginal ridge | 14. Point on the Distal outline corresponding to the midpoint of the Distal marginal ridge |
| 4.Lingual end of the distal marginal ridge: Junction of distal slope of lingual cusp and distal marginal ridge | 15. Distolingual line angle |
| 5.Tip of lingual cusp: Mesial and distal slopes of lingual cusp and triangular ridge | 16. Lingual crest of curvature |
| 6.Lingual end of the mesial marginal ridge: Junction of mesial slope of lingual cusp and mesial marginal ridge | 17. Mesiolingual line angle |
| 7.Mesial marginal ridge: mid point of the mesial marginal ridge | 18. Point on the Mesial outline corresponding to the midpoint of the Mesial marginal ridge |
| 8.Buccal end of the mesial marginal ridge: The point Junction of mesial slope of buccal cusp and mesial marginal ridge | 19. Mesiobuccal line angle |
| 9.Central groove point: junction of the buccal and lingual triangular ridges |  |
| 10.Distal triangular Pit: Junction of the central groove and the distal triangular fossa |  |
| 11.Mesial triangular Pit: Junction of the central groove and the mesial triangular fossa |  |

| Maxillary First/Second Molar |  |
| --- | --- |
| Landmarks by Anatomic Evidence | Landmarks by Geometric Evidence |
| 1.Mesiobuccal cusp tip – junction of the mesial and distal slopes of the mesiobuccal cusp and triangular ridge of the mesiobuccal cusp | 17.Buccal crest of curvature on mesiobuccal lobe |
| 2.Buccal groove point: junction of the distal slope of mesiobuccal cusp, mesial slope of distobuccal cusp and the buccal groove | 18.Termination of the buccal groove |
| 3.Distobuccal cusp tip: junction of the mesial and distal slopes of distobuccal cusp and triangular ridge of the distobuccal cusp | 19.Buccal crest of curvature on the Distobuccal lobe |
| 4.Buccal end of the distal marginal ridge: junction of the distal slope of distobuccal cusp and the distal marginal ridge. | 20.Distobuccal line angle |
| 5.Midpoint of the distal marginal ridge. | 21.Distal crest of curvature: distal most point on the distal surface |
| 6.Lingual end of the distal marginal ridge: junction of the distal slope of distolingual cusp and the distal marginal ridge | 22.Distopalatal line angle |
| 7.Distolingual cusp tip: mesial and distal cusp slopes of distolingual cusp and triangular ridge of distolingual cusp. | 23.Lingual crest of curvature on the distopalatal lobe |
| 8.Lingual groove point: junction of the distal slope of mesiolingual cusp, mesial slope of distolingual cusp and lingual groove | 24.Termination of the lingual/palatal groove |
| 9.Mesiolingual cusp tip: junction of the mesial and distal slopes of mesiolingual cusp and triangular ridges of mesiolingual cusp | 25.Lingual crest of curvature corresponding to the tip of the distopalatal cusp / cusp of carabelli if present |
| 10.Lingual end of the mesial marginal ridge: Junction of mesial slope of mesiolingual cusp and the mesial marginal ridge. | 26.Mesiopalatal line angle |
| 11.Midpoint of the mesial marginal ridge | 27.Mesial crest of curvature: Mesial most point on the mesial surface |
| 12.Buccal end of the mesial marginal ridge: junction of mesial cusp slope of mesiobuccal cusp and mesial marginal ridge | 28.Mesiobuccal line angle |
| 13.Distal triangular Pit: Junction of the central groove and the distal triangular fossa |  |
| 14.The midpoint of the groove crossing the oblique ridge |  |
| 15.Central Pit : meeting point of the buccal groove, Central Groove and the groove of the oblique ridge |  |
| 16.Mesial triangular Pit: Junction of the central groove and the mesial triangular fossa |  |

| Mandibular First Premolar |  |
| --- | --- |
| Landmarks by Anatomic Evidence | Landmarks by Geometric Evidence |
| 1.Buccal cusp tip- the point joining the mesial and the distal slopes of the buccal cusp and the buccal triangular ridge | 12.Buccal crest of curvature on the middle buccal lobe |
| 2.Buccal end of the distal marginal ridge: Junction of the distal marginal ridge and the distal cusp slope of the buccal cusp | 13.Distobuccal line angle |
| 3.Mid point of the distal marginal ridge | 14.Distal crest of curvature: a point of the distal outline of the tooth representing the distal most part of the distal outline |
| 4.Lingual end of the distal marginal ridge: Junction of the distal marginal ridge and the distal slope of the Lingual cusp | 15.Distolingual line angle |
| 5.Lingual cusp tip – the point joining the distal and the mesial slopes of the lingual cusp slopes and the triangular ridge of the lingual cusp | 16.Lingual crest of curvature on the lingual lobe |
| 6.Mesiolingual groove - The meeting point of the mesial slope of the lingual cusp, mesiolingual groove and the mesial marginal ridge | 17.Mesiolingual line angle |
| 7.Midpoint of the mesial marginal ridge | 18.End of the mesiolingual groove |
| 8.Buccal end of the mesial marginal ridge: junction of the mesial marginal ridge and the mesial slope of the buccal cusp | 19.Mesial crest of curvature: a point on the mesial outline of the tooth representing the mesial most part of the curvature |
| 9.Distal triangular pit: the junction of central groove and the distal triangular fossa | 20.Mesiobuccal line angle |
| 10.Meeting point of the buccal and lingual triangular ridge |  |
| 11.Mesial triangular pit: the junction of the central groove, mesiolingual groove and the end of the mesial triangular fossa |  |

| Mandibular Second Premolar (two cusp type) |  |
| --- | --- |
| Landmarks by Anatomic Evidence | Landmarks by Geometric Evidence |
| 1. Buccal cusp tip- the point joining the mesial & distal slopes of the buccal cusp and the buccal triangular ridge | 12. Buccal crest of curvature on the middle buccal lobe |
| 2. Buccal end of the distal marginal ridge: junction of the distal marginal ridge and the distal cusp slope of the buccal cusp | 13. Crest of curvature of the Distobuccal line angle |
| 3. Mid point of the distal marginal ridge | 14. Distal crest of curvature: a point on the distal most point on the distal outline of tooth |
| 4. Lingual end of the distal marginal ridge: junction of the distal marginal ridge and the distal slope of the lingual cusp | 15. Distolingual line angle |
| 5. Lingual cusp tip: junction of the mesial and distal slopes of the lingual cusp and the triangular ridge of the lingual cusp | 16. Lingual crest of curvature corresponding to the lingual lobe of the lingual cusp (two cusp type)/ end of the lingual groove (three cusp type) |
| 6. Lingual end of the mesial marginal ridge: the junction of the mesial slope of the lingual cusp (two cusp type)/mesiolingual cusp (three cusp type) and the mesial marginal ridge | 17. Mesiolingual line angle |
| 7. Midpoint of the mesial marginal ridge | 18. Mesial crest of curvature: the most mesial point on the mesial outline of the tooth |
| 8. Buccal end of the mesial marginal ridge: junction of the mesial marginal ridge and the mesial slope of the buccal cusp | 19. Mesiobuccal line angle |
| 9. Distal triangular pit: junction of the central groove and the distal triangular fossa |  |
| 10. Central Pit: the lingual most part of the central groove at the junction of the buccal and lingual triangular ridges |  |
| 11. Mesial triangular pit: junction of the central groove and the mesial triangular fossa. |  |

| Mandibular Second Premolar (3 cusp type) |  |
| --- | --- |
| Landmarks by Anatomic Evidence | Landmarks by Geometric Evidence |
| 1. Buccal cusp tip- the point joining the mesial & distal slopes of the buccal cusp and the buccal triangular ridge | 14. Buccal crest of curvature on the middle buccal of the lobe |
| 2. Buccal end of the distal marginal ridge: junction of the distal marginal ridge and the distal cusp slope of the buccal cusp | 15. Crest of curvature on the Distobuccal line angle |
| 3. Mid point of the distal marginal ridge | 16. Distal crest of curvature: a point on the distal most point on the distal outline of tooth |
| 4. Lingual end of the distal marginal ridge: junction of the distal marginal ridge and the distal slope of the distolingual cusp (three cusp type)/ lingual cusp (two cusp type) | 17. Crest of curvature on the Distolingual line angle |
| 5. Distolingual cusp tip: junction of the mesial and distal slope of the distolingual cusp and the triangular ridge of the distolingual cusp | 18. Crest of curvature on the lingual surface corresponding to direction/crossing of the lingual groove |
| 6. Lingual groove: the junction of the mesial slope of the distolingual cusp and the distal slope of the mesiolingual cusp and the lingual groove | 19. Crest of curvature on the Mesiolingual line angle |
| 7. Mesiolingual cusp tip: junction of the mesial and distal slope of the mesiolingual cusp and the triangular ridge of the mesiolingual cusp | 20. Mesial crest of curvature: the most mesial point on the mesial outline of the tooth |
| 8. Lingual end of the mesial marginal ridge: the junction of the mesial slope of the lingual cusp (two cusp type)/mesiolingual cusp (three cusp type) and the mesial marginal ridge | 21. Crest of curvature on the Mesiobuccal line angle |
| 9. Midpoint of the mesial marginal ridge |  |
| 10. Buccal end of the mesial marginal ridge: junction of the mesial marginal ridge and the mesial slope of the buccal cusp |  |
| 11. Distal triangular pit: junction of the central groove and the distal triangular fossa |  |
| 12. Central pit, In three cusp type : junction of the central groove and the lingual groove |  |
| 13. Mesial triangular pit: junction of the central groove and the mesial triangular fossa. |  |

| Mandibular First Molar |  |
| --- | --- |
| Landmarks by Anatomic Evidence | Landmarks by Geometric Evidence |
| 1. Mesiobuccal cusp tip – mesial and distal slope of mesiobuccal cusp and triangular ridge | 20. Buccal crest of curvature corresponding to mesiobuccal cusp tip |
| 2. Mesiobuccal groove point: distal slope of mesiobuccal cusp mesial slope of distobuccal cusp and mesiobuccal groove junction. | 21. End of the mesiobuccal groove on the buccal surface |
| 3. Distobuccal cusp tip: mesial and distal slope of distobuccal cusp and triangular ridge of the distobuccal cusp | 22. Buccal crest of curvature corresponding to the Distobuccal cusp tip |
| 4. Distobuccal groove point: distal slope of the distobuccal cusp, mesial slope of the distal cusp and the distobuccal groove junction. | 23. End of the distobuccal groove on the buccal surface |
| 5. Distal cusp tip: junction of the mesial and distal slope of the distal cusp and the triangular ridge of the distal cusp | 24. Distobuccal line angle |
| 6. Buccal end of the distal marginal ridge: Junction of the distal slope of distal cusp and distal marginal ridge. | 25. Point corresponding to the midpoint of the distal marginal ridge |
| 7. Midpoint of the distal marginal ridge. | 26. Distolingual line angle |
| 8. Lingual end of the distal marginal ridge: Distal slope of distolingual cusp and distal marginal ridge. | 27. Lingual crest of curvature corresponding to the distolingual cusp tip |
| 9. Distolingual cusp tip: mesial and distal cusp slopes and triangular ridge of distolingual cusp. | 28. End of the lingual groove on the lingual surface |
| 10. Lingual groove point: distal slope of mesiolingual cusp, mesial slope of distolingual cusp and lingual groove. | 29. Lingual crest of curvature corresponding to the distolingual cusp |
| 11. Mesiolingual cusp tip: Mesial and distal slope and mesiolingual cusp and triangular ridge of mesiolingual cusp | 30. Mesiolingual line angle |
| 12. Lingual end of the mesial marginal ridge : Junction of mesial slope of mesiolingual cusp and mesial marginal ridge. | 31. Point corresponding to the midpoint of the mesial marginal ridge |
| 13. Midpoint of the mesial marginal ridge | 32. Mesiobuccal line angle |
| 14. Buccal end of the mesial marginal ridge: junction of mesial cusp slope of mesiobuccal cusp and mesial marginal ridge. |  |
| 15. Distal pit: Distal end of the central groove which bifurcates to the distal triangular fossa |  |
| 16. Junction of the distobuccal groove to the central groove |  |
| 17. Junction of the lingual groove to the central groove (49) |  |
| 18. Central Pit : meeting point of the mesiobuccal groove, lingual groove(sometimes) and the Central Groove |  |
| 19. Mesial Pit: Mesial end of the central groove which bifurcates to enclose the mesial triangular fossa |  |

| Mandibular second molar |  |
| --- | --- |
| Landmarks by Anatomic Evidence | Landmarks by Geometric Evidence |
| 1. Mesiobuccal cusp tip – mesial and distal slope of mesiobuccal cusp and triangular ridge | 16. Buccal crest of curvature corresponding to mesiobuccal cusp tip |
| 2. Buccal groove point: distal slope of mesiobuccal cusp mesial slope of distobuccal cusp and mesiobuccal groove junction. | 17. End of the buccal groove on the buccal surface |
| 3. Distobuccal cusp tip: mesial and distal slope of distobuccal cusp and triangular ridge of the distobuccal cusp | 18. Buccal crest of curvature corresponding to the Distobuccal cusp tip |
| 4. Buccal end of the distal marginal ridge: Junction of the distal slope of distobuccal cusp and distal marginal ridge. | 19. Distobuccal line angle |
| 5. Midpoint of the distal marginal ridge. | 20. Point corresponding to the midpoint of the distal marginal ridge |
| 6. Lingual end of the distal marginal ridge: Distal slope of distolingual cusp and distal marginal ridge. | 21. Distolingual line angle |
| 7. Distolingual cusp tip: mesial and distal cusp slopes and triangular ridge of distolingual cusp. | 22. Lingual crest of curvature corresponding to the distolingual cusp tip |
| 8. Lingual groove point: distal slope of mesiolingual cusp, mesial slope of distolingual cusp and lingual groove. | 23. End of the lingual groove on the lingual surface |
| 9. Mesiolingual cusp tip: Mesial and distal slope and mesiolingual cusp and triangular ridge of mesiolingual cusp | 24. Lingual crest of curvature corresponding to the distolingual cusp |
| 10. Lingual end of the mesial marginal ridge : Junction of mesial slope of mesiolingual cusp and mesial marginal ridge. | 25. Mesiolingual line angle |
| 11. Midpoint of the mesial marginal ridge | 26. Point corresponding to the midpoint of the mesial marginal ridge |
| 12. Buccal end of the mesial marginal ridge: junction of mesial cusp slope of mesiobuccal cusp and mesial marginal ridge. | 27. Mesiobuccal line angle |
| 13. Distal pit: Distal end of the central groove which bifurcates to form the distal triangular fossa |  |
| 14. Central Pit : meeting point of the mesiobuccal groove, lingual groove and the Central Groove |  |
| 15. Mesial Pit: Mesial end of the central groove which bifurcates to enclose the mesial triangular fossa |  |
